# Kidney function and other factors and their association with falls: The screening for CKD among older people across Europe (SCOPE) study

**DOI:** 10.1186/s12877-020-01698-2

**Published:** 2020-10-02

**Authors:** Sabine Britting, Rada Artzi-Medvedik, Paolo Fabbietti, Lisanne Tap, Francesco Mattace-Raso, Andrea Corsonello, Fabrizia Lattanzio, Johan Ärnlöv, Axel C. Carlsson, Regina Roller-Wirnsberger, Gerhard Wirnsberger, Tomasz Kostka, Agnieszka Guligowska, Francesc Formiga, Rafael Moreno-Gonzalez, Pedro Gil, Sara Lainez Martinez, Robert Kob, Itshak Melzer, Ellen Freiberger, on behalf of the SCOPE investigators, Fabrizia Lattanzio, Andrea Corsonello, Silvia Bustacchini, Silvia Bolognini, Paola D’Ascoli, Raffaella Moresi, Giuseppina Di Stefano, Cinzia Giammarchi, Anna Rita Bonfigli, Roberta Galeazzi, Federica Lenci, Stefano Della Bella, Enrico Bordoni, Mauro Provinciali, Robertina Giacconi, Cinzia Giuli, Demetrio Postacchini, Sabrina Garasto, Annalisa Cozza, Francesco Guarasci, Sonia D’Alia, Romano Firmani, Moreno Nacciariti, Mirko Di Rosa, Paolo Fabbietti

**Affiliations:** 1 Department of Internal Medicine-Geriatrics, Institute for Biomedicine of Aging (IBA), Friedrich-Alexander-Universität Erlangen-Nürnberg, Erlangen, Germany; 2 Department of Nursing, Recanati School for Community Health Professions at the faculty of Health Sciences, Ben-Gurion University of the Negev, Beersheba, Israel; 3 Italian National Research Center on Aging (IRCCS INRCA), Fermo and Cosenza, Ancona, Italy; 4 Laboratory of Geriatric Pharmacoepidemiology and Biostatistics, IRCCS INRCA, Via S. Margherita 5, 60124 Ancona, Italy; 5 Department of Internal Medicine, Section of Geriatric Medicine, Erasmus University Medical Center Rotterdam, Rotterdam, The Netherlands; 6 Department of Medical Sciences, Uppsala University, Uppsala, Sweden; 7 Division of Family Medicine, Department of Neurobiology, Care Sciences and Society, Karolinska Institutet, Stockholm, Sweden; 8 School of Health and Social Studies, Dalarna University, Falun, Sweden; 9 Department of Internal Medicine-Geriatrics, Medical University of Graz, Graz, Austria; 10 Division of Nephrology, Department of Internal Medicine, Medical University of Graz, Graz, Austria; 11 Department of Geriatrics, Healthy Ageing Research Centre, Medical University of Lodz, Lodz, Poland; 12 Geriatric Unit, Internal Medicine Department, Bellvitge University Hospital – IDIBELL – L’Hospitalet de Llobregat, Barcelona, Spain; 13 Geriatric Department, Hospital Clínico San Carlos, Martín Lagos S/N, 28040 Madrid, Spain; 14 Department of Physical Therapy, Recanati School for Community Health Professions at the faculty of Health Sciences, Ben-Gurion University of the Negev, Beersheba, Israel

**Keywords:** Kidney function, Older people, Falls, Injurious falls, Fear of falling, Urinary incontinence

## Abstract

**Background:**

Reduced kidney function has become a major public health concern, especially among older people, as Chronic Kidney Disease (CKD) is associated with increased risk of end stage renal disease and mortality. Falls are a serious negative health outcome in older persons with one third of people aged 65 years experiencing a fall per year and increasing fall rates with increasing age. The impact of CKD on falls in older community-dwelling persons is not well investigated. Additionally, lower urinary tract symptoms (LUTS) may also increase the risk of falls. Therefore, our aim was to investigate the impact of CKD and LUTS on falls as well as on injurious falls.

**Methods:**

The SCOPE study is an observational, multinational, multicenter, prospective cohort study involving community-dwelling older persons aged 75 years and more recruited from August 2016 to March 2018 in seven European countries. The main outcomes of the present study were any falls and any injurious falls during the 12 months before enrolment. The cross-sectional association of estimated glomerular filtration rate (eGFR) and LUTS with study outcomes was investigated by logistic regression analysis adjusted for baseline characteristics of enrolled subjects.

**Results:**

Our series consisted of 2256 SCOPE participants (median age = 79.5 years, 55.7% female). Of them, 746 participants experienced a fall and 484 reported an injurious fall in the 12 months prior to baseline assessment. CKD was not significantly associated with falls (*OR* = 0.95, 95%*CI* = 0.79–1.14 for eGFR< 60; *OR* = 1.02, 95%*CI* = 0.81–1.28 for eGFR< 45; *OR* = 1.08, 95%*CI* = 0.74–1.57 for eGFR< 30) or injurious falls (*OR* = 0.91, 95%*CI* = 0.67–1.24 for eGFR< 60; *OR* = 0.93, 95%*CI* = 0.63–1.37 for eGFR< 45; *OR* = 1.19, 95%*CI* = 0.62–2.29 for eGFR< 30). LUTS were found significantly associated with both falls (*OR* = 1.56, 95%*CI* = 1.29–1.89) and injurious falls (*OR* = 1.58, 95%*CI* = 1.14–2.19), and such associations were confirmed in all multivariable models.

**Conclusions:**

Cross-sectional data suggest that CKD may not be associated with history of falls or injurious falls, whereas LUTS is significantly associated with the outcomes.

**Trial registration:**

This study was registered on 25th February 2016 at clinicaltrials.gov (NCT02691546).

## Background

Due to the demographic changes, new challenges arise for the public health care systems and in older persons themselves. Current clinical and public health approaches more and more try to sustain functional capacity and resilience, including functionality in an integrated care concept for all citizens and across lifespan. Major endpoints of adverse life trajectories are deteriorating chronic illnesses and geriatric syndromes. A fall or injurious fall threatens functionality and independence in older persons and are often the start of a downward spiral leading to nursing home admission or even death. Therefore, the International Consortium for Health Outcomes Measurement (ICHOM) included “falls” as a standard set to determine health outcome [1].

One third of persons older than 65 years experienced at least one fall per year and history of falling doubles the chances of additional falls [2]. Among community-dwelling people aged 80 or more the percentage of fallers increases up to 50% [3, 4]. Very often falls -based on different definitions- are accompanied by fractures [5], causing distress, pain and immobilization. In the US overall medical costs for fatal falls was estimated to be $754 million [5]. As the older population still increases, the number of fall injuries and the cost to treat them will pose a tremendous burden to the health care systems.

Many falls do not cause injuries, but they have negative impact on quality of life [6] and independency in daily activities [7–9]. In the event of a fall, self-efficacy can decrease due to fall-related psychological concerns (FrPC). It has been reported earlier, that 21 and 85% of older adults suffer from FrPC, which may be seen as an independent risk factor for falls [10, 11]. FrPC may lead to inactivity, decreased physical function, symptoms of depression and therefore reduced quality of life [12]. By nature, falls are defined as geriatric syndromes with a multi-causal nature. In this context, frailty, polypharmacy and chronic diseases, such as chronic kidney disease (CKD) have also been identified as factors promoting risk of falling [13].

Information on the impact of CKD on falls in older community-dwelling persons are rare. Among people aged 65 or more, Kistler et al. [14] demonstrated an increased odds ratio (1.81) for falls in CKD patients. This finding was confirmed by Paliwal et al. [15] in an US Population with an adjusted odds ratio of 1.27 (95% CI 1.14–1.42). In the above studies blood and urine analysis were not performed, eGFR were not reported, and diagnosis of CKD was only based on clinical history.

Lower urinary tract symptoms (LUTS) also deserve to be considered when investigating the association between CKD and falls. In a recent small study by Lai et al. results demonstrated an elevated prevalence of urological diseases [16] in CKD older persons. Different pathways have been proposed for this relationship including medication as diuretics for hypertension, overactive bladder especially for women, or changes in the bladder urothelium [17]. Additionally, urinary incontinence has been demonstrated as a risk factor on falls [18, 19].

In this context, the SCOPE study provides a clinically relevant source of community-dwelling older adults to study the relationship between chronic diseases, such as CKD, functionality and the risk of falling in a population older than 75 years. The objectives of the present investigation were (1) to evaluate different intrinsic correlates of history of falling based upon self-report from participants at baseline; (2) to evaluate correlates of history of injurious falls at baseline retrospectively; (3) to explore the impact of CKD and LUTS on falls and injurious falls.

## Methods

### Study design and participants

The SCOPE study (European Grant Agreement no. 436849), is a multicenter 2-year prospective cohort study involving patients older than 75 years attending outpatient services in participating institutions in seven countries (Austria, Germany, Israel, Italy, the Netherlands, Poland and Spain). Methods of the SCOPE study have been extensively described elsewhere. Participants were requested to sign a written informed consent before entering the study. The study protocol was approved by ethics committees at all participating institutions, and complies with the Declaration of Helsinki and Good Clinical Practice Guidelines. Only baseline data are used in the present study. Exclusion criteria were: end-stage renal disease or dialysis at time of enrollment; history of solid organ or bone marrow transplantation; active malignancy within 24 months prior to screening or metastatic cancer; life expectancy less than 6 months (based on the judgment of the study physician after careful medical history collection and diagnoses emerging from examination of clinical documentation exhibited); severe cognitive impairment (Mini Mental State Examination < 10); any medical or other reason (e.g. known or suspected patients’ inability to comply with the protocol procedure) in the judgement of the investigators, that the patient was unsuitable for the study; unwilling to provide consent and limited possibility to attend follow-up visits [20].

Overall, 2461 participants were initially enrolled in the study. Of them, 2256 participants were included in the analysis presented in the current publication and 205 participants had to be excluded from analysis due to incomplete baseline data collected between August 2016 to March 2018.

### Measures

#### Sociodemographic, anthropometry, laboratory analysis

Data on history of falls and incident falls were collected retrospective via a face-to face interview questionnaire. For prevalence of falls and injurious falls, two questions were asked: 1) how many times have you fallen in the past 12 months, (answer “No” for 0 events, and the answer “yes” for 1 or more falls), and 2) what kind of injuries did you sustain from a fall (answer: (fractures, treated and untreated injury, and no injury). Further information of the location and circumstances of the fall were documented. An injurious fall was defined as a fall causing fractures, treated or untreated injuries. Fear of falling (FoF) was obtained with a single question with possible answers ranging from not at all concerned, somewhat concerned, fairly concerned and very concerned.

To assess the CKD stage, blood and urine analysis were performed by the locally certified laboratories adhering to the protocol. Serum creatinine was measured at local level by standard methods. Creatinine-based eGFR was calculated using the Berlin Initiative Study 1 (BIS1) equation, especially developed for persons 70 years and older [21] using serum creatinine levels. Three different severity threshold for CKD were used: < 60, < 45 and < 30 ml/min/1.73m^2^.

Lower urinary tract symptoms (LUTS) were assessed by asking the patient to rate on a 5 Likert point scale (0–4) the presence of LUTS within the last 4 weeks [22]. Urinary incontinence was defined as at least one moderate or big problem in dripping or leaking urine, weak urine stream or incomplete emptying, waking up to urinate, need to urinate frequently during the day.

Participant’s characteristics were assessed during the baseline interview and medical examination. Demographic variables included age, gender and self-reported living-status (living alone vs. living with others). Body mass index (BMI) was calculated by body weight and height and expressed as kg/m^2^, measured according to standard operating procedures.

Comprehensive geriatric assessment (CGA) was performed including Mini Mental State Examination (MMSE)/cognitive status [23], 15-items Geriatric Depression Scale (GDS)/mood [24], Basic (ADL) and Instrumental Activities of Daily Living (IADL)/self-reported disability [25, 26]. Physical function was assessed with the short physical performance battery (SPPB) [27] including gait speed, five chair-stands test (time to rise from a chair and return to the seated position 5 times without using arms) and balance test (ability to stand with the feet together in the side-by-side, semi-tandem, and tandem positions).

Diseases were documented and analyzed using Cumulative Illness Rating Scale for Geriatrics (CIRS-G Total Score)/overall comorbidity [28]. The number of medications was also calculated and included in the analysis.

Health related quality of life was rated by Euro-Qol 5D [29] questionnaire, including total score and QoL visual analogue scale (EQ-VAS).

### Statistical analysis

First, we compared participants grouped according to self-reported falls or injurious falls during the 12 months before enrolment. Chi-square test was used to compare categorical variables and Student T-test for continuous ones. In order to obtain an adjusted estimate of the association of eGFR or LUTS with study outcomes, we built logistic regression models adjusted for age, gender, SPPB score, GDS, IADL, Euro-Qol 5D. The analyses were also repeated by removing SPPB from multivariable models.

Statistical analysis was carried out using SPSS for Win V24.0 (SPSS Inc., Chicago, IL, USA). *p*-value of < 0.05 was considered statistically significant.

## Results

Overall, 2256 participants were included in the analysis (median age 79.5 years (5.9 interquartile range (IR), and 55.7% female) (Table 1). At least 1 fall was reported by 746 (33.1%) participants. Of them, 484 (64.9%) reported an injurious fall and 262 reported a non-injurious fall in the 12 months prior to baseline assessment.

**Table 1:** Characteristics of the study population in total, in fallers and non-fallers reported retrospectively in the past year

Variable	All (*N* = 2256)	No falls past 12 months (*N* = 1510)	At least 1 fall past 12 months (*N* = 746)	*p*
Age, years	**79.5 (5.9)**	**79.4 (5.5)**	**79.6 (6.6)**	**0.022**
Gender (Female), n (%)	**1256 (55.7)**	**795 (52.6)**	**461 (61.8)**	**< 0.001**
Educational level, years	11.0 (7.0)	11.0 (7.0)	11.0 (6.0)	0.973
Living alone, n (%)	**551 (24.4)**	**341 (22.6)**	**210 (28.2)**	**0.004**
MMSE	29.0 (3.0)	29.0 (3.0)	29.0 (3.0)	0.212
GDS	**2.0 (3.0)**	**2.0 (3.0)**	**2.0 (3.0)**	**< 0.001**
IADL	**2.0 (9.0)**	**2.0 (8.0)**	**3.0 (11.0)**	**< 0.001**
Urinary incontinence (LUTS), n (%)	**653 (28.9)**	**390 (25.8)**	**263 (35.3)**	**< 0.001**
Euro-Qol 5D				
Score	**7.0 (4.0)**	**7.0 (3.0)**	**8.0 (4.0)**	**< 0.001**
"your health today”	**73.0 (20.0)**	**75.0 (25.0)**	**70.0 (30.0)**	**< 0.001**
CIRS-G Total Score	**8.0 (6.0)**	**8.0 (6.0)**	**8.0 (7.0)**	**< 0.001**
Comorbidities				
*Hypertension*, n (%)	1733 (76.8)	1147 (76.0)	586 (78.6)	0.170
*Stroke*, n (%)	**131 (5.8)**	**68 (4.5)**	**63 (8.4)**	**< 0.001**
*Hip fracture*, n (%)	**111 (4.9)**	**49 (3.2)**	**62 (8.3)**	**< 0.001**
*Osteoporosis*, n (%)	**688 (30.5)**	**423 (28.0)**	**265 (35.5)**	**< 0.001**
*Parkinson*, n (%)	45 (2.0)	25 (1.7)	20 (2.7)	0.101
*Diabetes*, n (%)	568 (25.2)	369 (24.4)	199 (26.7)	0.249
Number of medications	**4.0 (8.0)**	**4.0 (7.0)**	**5.0 (8.0)**	**< 0.001**
eGFR (BIS equation)				0.586
*≥ 60*, n (%)	832 (36.9)	551 (36.5)	281 (37.7)	
*< 60*, n (%)	1424 (63.1)	959 (63.5)	465 (62.3)	
***< 45, n (%)***	***561 (24.9)***	***369 (24.2)***	***192 (25.7)***	***0.862***
***< 30, n (%)***	***141 (6.3)***	***91 (6.0)***	***50 (6.7)***	***0.696***
BMI, kg/m^2^	27.3 (5.7)	27.3 (5.6)	27.3 (6.2)	0.939
SPPB Total Score	**9.0 (4.0)**	**10.0 (3.0)**	**9.0 (5.0)**	**< 0.001**
SPPB Balance				**< 0.001**
*Severe limitation*, n (%)	**432 (20.1)**	**241 (16.6)**	**191 (27.4)**	
*Moderate limitation*, n (%)	**410 (19.1)**	**265 (18.3)**	**145 (20.8)**	
*No limitation*, n (%)	**1307 (60.8)**	**946 (65.2)**	**361 (51.8)**	
SPPB Gait Speed				**< 0.001**
*≤ 8.70 s*, n (%)	**2040 (92.2)**	**1393 (93.7)**	**647 (89.0)**	
*> 8.70 s*, n (%)	**173 (7.8)**	**93 (6.3)**	**80 (11.0)**	

Note: values are expressed as percentage or median (IQR)

*Abbreviations*: *ADL* activities of daily living, *BIS* Berlin Initiative Study, *BMI* body mass index, *CIRS-G* cumulative illness rating scale for geriatrics, *eGFR* estimated glomerular filtration rate, *GDS* geriatric depression scale, *IADL* instrumental activities of daily living, *LUTS* lower urinary tract symptoms, *MMSE* mini mental state examination, *SSPB* short physical performance battery

### Fallers versus non-fallers

Fallers were older and more frequently female compared to non-fallers. Living alone was more prevalent among fallers, who also exhibited higher number of medications, IADL score and Euro-Qol 5D Total score, as well as lower SPPB Total Score. LUTS were significantly more frequent in fallers than in non-fallers, while no significant difference was observed in regards to kidney function (Table 1).

### Injurious fallers versus non-injurious fallers

Female gender and LUTS were more frequent among injurious vs non-injurious fallers. Injurious fallers also had higher Geriatric Depression Scale and IADL score, while SPPB Total score was lower in injurious vs non-injurious fallers. Even in this case, no significant difference was observed in regards to kidney function. Fair concern for falling was more frequent among injurious fallers (Table 2).

**Table 2:** Characteristics of the study population and comparisons between retrospectively reported injurious falls versus non-injurious falls

Variable	All (*N* = 746)	No injurious falls (Fractures/treated injury/untreated injury) (*N* = 262)	At least 1 injurious fall (Fractures/treated injury/untreated injury) (*N* = 484)	*p*
Age, yrs.	79.6 (6.6)	80.0 (6.6)	79.5 (6.7)	0.259
Gender (Female), n (%)	**461 (61.8)**	**140 (53.4**	**321 (66.3)**	**0.001**
Educational level, years	11.0 (6.0)	11.0 (6.0)	11.0 (7.0)	0.569
Living alone, n (%)	210 (28.2)	71 (27.1)	139 (28.7)	0.639
MMSE score	29.0 (3.0)	29.0 (3.0)	29.0 (3.0)	0.947
Geriatric Depression Score	**2.0 (3.0)**	**2.0 (3.0)**	**3.0 (4.0)**	**0.001**
IADL	**3.0 (11.0)**	**2.0 (10.0)**	**4.0 (11.0)**	**0.046**
Urinary incontinence (LUTS), n (%)	**263 (35.3)**	**75 (28.6)**	**188 (38.8)**	**0.005**
Euro-Qol 5D				
Total Score	**8.0 (4.0)**	**8.0 (4.0)**	**8.0 (5.0)**	**0.002**
"your health today”	70.0 (30.0)	70.0 (20.0)	70.0 (30.0)	0.056
CIRS-G Total Score	8.0 (7.0)	8.0 (7.0)	9.0 (7.0)	0.545
Comorbidities				
*Hypertension*, n (%)	586 (78.6)	197 (75.2)	389 (80.4)	0.100
*Stroke*, n (%)	63 (8.4)	16 (6.1)	47 (9.7)	0.091
*Hip fracture*, n (%)	**62 (8.3)**	**11 (4.2)**	**51 (10.5)**	**0.003**
*Osteoporosis*, n (%)	**265 (35.5)**	**74 (28.2)**	**191 (39.5)**	**0.002**
*Parkinson*, n (%)	20 (2.7)	7 (2.7)	13 (2.7)	0.991
*Diabetes*, n (%)	199 (26.7)	76 (29.0)	123 (25.4)	0.289
Number of medications	5.0 (8.0)	5.0 (8.0)	5.0 (8.0)	0.967
eGFR (BIS equation)				0.559
*≥ 60*, n (%)	281 (37.7)	95 (36.3)	186 (38.4)	
*< 60*, n (%)	465 (62.3)	167 (63.7)	298 (61.6)	
***< 45, n (%)***	**192 (25.7)**	**68 (26.0)**	**124 (25.6)**	**0.718**
***< 30, n (%)***	**50 (6.7)**	**15 (5.7)**	**35 (7.2)**	**0.598**
BMI, kg/m^2^	27.3 (6.2)	27.1 (6.4)	27.3 (6.0)	0.877
SPPB Total Score	**9.0 (5.0)**	**9.0 (5.0)**	**8.5 (6.0)**	**0.032**
SPPB Balance				0.184
*Severe limitation*, n (%)	191 (27.4)	56 (23.1)	135 (29.7)	
*Moderate limitation*, n (%)	145 (20.8)	53 (21.9)	92 (20.2)	
*No limitation*, n (%)	361 (51.8)	133 (55.0)	228 (50.1)	
SPPB Gait Speed				**0.026**
*≤ 8.70 s*, n (%)	**647 (89.0)**	**235 (92.5)**	**412 (87.1)**	
*> 8.70 s*, n (%)	**80 (11.0)**	**19 (7.5)**	**61 (12.9)**	
Fear of falling				
Single question: not at all concerned	**337 (45.1)**	**141 (53.6)**	**196 (40.5)**	**0.001**
Single question: fairly concerned	**118 (15.8)**	**26 (9.9)**	**92 (19.0)**	**0.001**

Note: values are expressed as percentage or median (IQR)

*Abbreviations*: *ADL* activities of daily living, *BIS* Berlin Initiative Study, *BMI* body mass index, *CIRS-G* cumulative illness rating scale for geriatrics, *eGFR* estimated glomerular filtration rate, *GDS* geriatric depression scale, *IADL* instrumental activities of daily living, *LUTS* lower urinary tract symptoms, *MMSE* mini mental state examination, *SSPB* short physical performance battery

### Multivariable analysis

In logistic regression analyses models no significant association was found between CKD and any falls (Table 3). The lack of significant association between CKD and falls was also confirmed when considering different CKD stages (e.g. < 30 or < 45 ml/min/1.73 m^2^) (Table 3). In model 2, female gender was significantly associated with the occurrence of any falls (*OR* = 1.45, 95%*CI* = 1.21–1.74). Such an association was also observed in all multivariable models (data not shown). Other significant correlates of the occurrence of any falls were SPPB (*OR* = 0.89, 95%*CI* = 0.86–0.92) in model 3, incontinence (*OR* = 1.37, 95%*CI* = 1.12–1.67) in model 4, and incontinence (*OR* = 1.33, 95%*CI* = 1.09–1.63), quality of life (*OR* = 1.03, 95%*CI* = 1.03–1.10), and IADL (*OR* = 1.02, 95%*CI* = 1.0–1.03). Similarly, CKD was not associated with injurious falls. Even in this case female gender was significantly associated with the outcome (*OR* = 1.70, 95%*CI* = 1.25–2.31) in model 2, and this finding was confirmed in all multivariable models (data not shown). Other significant predictors of injurious falls were SPPB (*OR* = 0.95, 95%*CI* = 0.90–1.0) in model 3, incontinence (*OR* = 1.46, 95% = 1.04–2.05), GDS (*OR* = 1.09, 95%*CI* = 1.02–1.17) and IADL (*OR* = 1.03, 95%*CI* = 1.0–1.06) in model 4. Predictors in model 5 were almost identical to those in model 4 (data not shown). Notably, patients with more severe stages of CKD were older and more frequently male, had greater IADL dependency, CIRS-G score and number of medications, as well as lower SPPB total score and higher prevalence of hypertension, stroke and osteoporosis (Table 4).

**Table 3:** Probability of at least 1 fall (*n* = 2256) and of at least 1 injurious fall (*n* = 746) in CKD groups, in the SCOPE cohort at baseline

Predictors	OR (95% CI) At least 1 fall	OR (95% CI) At least 1 injurious fall
**Model 1.** Compares CKD group (combining several stages in CKD) vs. non-CKD group	0.95 (0.79–1.14)	0.91 (0.67–1.24)
**Model 1.2 Compares CKD group (< 45) vs. non-CKD group**	**1.02 (0.81–1.28)**	**0.93 (0.63–1.37)**
**Model 1.3 Compares CKD group (< 30) vs. non-CKD group**	**1.08 (0.74–1.57)**	**1.19 (0.62–2.29)**
**Model 2.** Adjusted for age and gender	0.91 (0.75–1.10)	0.97 (0.70–1.34)
**Model 3.** Adjusted for age, gender and SPPB total score	0.84 (0.69–1.01)	0.93 (0.67–1.28)
**Model 4.** Adjusted for age, gender, SPPB total score, GDS, incontinence, IADL score and Euro-Qol 5D Score	0.84 (0.69–1.02)	0.92 (0.66–1.28)
**Model 5.** Adjusted for age, gender, GDS, incontinence, IADL score and Euro-Qol 5D Score	0.87 (0.72–1.05)	^a^0.94 (0.68–1.32)

Note: ^a^2 “fear of falling” variables were added (not at all concerned vs other; fairly concerned vs other)

*Abbreviations*: *CI* confidence interval, *CKD* chronic kidney disease, *GDS* geriatric depression score, *IADL* instrumental activities of daily living, *OR* odds ratio, *SPPB* short physical performance battery

**Table 4:** Significant correlates of eGFR categories in the patients studied

Variable	eGFR > 60 *N* = …	eGFR 45–60 *N* = …	eGFR 30–45 *N* = …	eGFR < 30 *N* = …	*p*
**Age, years**	**78.6 (4.5)**	**79.6 (5.7)**	**80.9 (6.8)**	**81.0 (7.2)**	**< 0.001**
**Gender (Female), n (%)**	**508 (61.1)**	**490 (56.8)**	**201 (47.9)**	**57 (40.4)**	**< 0.001**
**IADL**	**0.0 (6.0)**	**2.0 (8.0)**	**6.0 (12.0)**	**12.0 (14.0)**	**< 0.001**
**CIRS-G Total Score**	**7.0 (6.0)**	**7.0 (5.0)**	**10.0 (7.0)**	**11.5 (7.2)**	**< 0.001**
Comorbidities					
***Hypertension*, n (%)**	**552 (66.3)**	**670 (77.6)**	**375 (89.3)**	**136 (96.5)**	**< 0.001**
***Stroke*, n (%)**	**36 (4.3)**	**49 (5.7)**	**33 (7.9)**	**13 (9.2)**	**0.022**
***Osteoporosis*, n (%)**	**247 (29.7)**	**255 (29.5)**	**122 (29.0)**	**64 (45.4)**	**0.001**
*Diabetes*, n (%)	**162 (19.5)**	**195 (22.6)**	**160 (38.1)**	**51 (36.2)**	**< 0.001**
**Number of medications**	**2.0 (6.0)**	**4.0 (7.0)**	**6.0 (7.0)**	**7.0 (5.0)**	**< 0.001**
**SPPB Total Score**	**10.0 (3.0)**	**10.0 (4.0)**	**8.0 (6.0)**	**7.0 (5.0)**	**< 0.001**

Note: values are expressed as percentage or median (IQR)

*Abbreviations*: *CIRS-G* cumulative illness rating scale for geriatrics, *eGFR* estimated glomerular filtration rate, *IADL* instrumental activities of daily living, *SPPB* short physical performance battery

Urinary incontinence (defined by the LUTS) was found associated with both any and injurious falls in all multivariable models (Table 5). Other predictors of any falls in this analysis were female gender (*OR* = 1.42, 95%*CI* = 1.19–1.71) in model 2, SPPB (*OR* = 0.90, 95%*CI* = 0.87–0.93) in model 3, SPPB (*OR* = 0.92, 95%*CI* = 0.88–0.95) in model 4, quality of life (*OR* = 1.06, 95%*CI* = 1.03–1.10) and IADL (*OR* = 1.02, 95%*CI* = 1.0–1.03) in model 5. Female gender was significantly associated with any falls in all multivariable models (data not shown). Female gender (*OR* = 1.66, 95%*CI* = 1.21–2.26) was significantly associated with injurious falls in model 2, and such association was confirmed in all multivariable models (data not shown). Finally, GDS (*OR* = 1.09, 95%*CI* = 1.02–1.17) and IADL (*OR* = 1.03, 95%*CI* = 1.0–1.06) also qualified as significant correlates of injurious falls in model 4, and almost identical findings were observed in model 5 (data not shown).

**Table 5:** Probability of at least 1 fall (*n* = 2256) and of at least 1 injurious fall (*n* = 746) in continence/incontinence groups, in the SCOPE cohort at baseline

Predictors	OR (95% CI) At least 1 fall	OR (95% CI) At least 1 injurious fall
**Model 1.** Compares incontinence group (at least 1 moderate or big problem in dripping or leaking urine, weak urine stream or incomplete emptying, waking up to urinate, need to urinate frequently during the day) vs. non incontinence group (other)	**1.56 (1.29–1.89)**	**1.58 (1.14–2.19)**
**Model 2.** Adjusted for age and gender	**1.51 (1.25–1.83)**	**1.55 (1.12–2.15)**
**Model 3.** Adjusted for age, gender and SPPB total score	**1.46 (1.20–1.77)**	**1.52 (1.10–2.12)**
**Model 4.** Adjusted for age, gender, SPPB total score, GDS, CKD, IADL score and Euro-Qol 5D Score	**1.37 (1.11–1.67)**	**1.46 (1.04–2.05)**
**Model 5.** Adjusted for age, gender, GDS, CKD, IADL score and Euro-Qol 5D Score	**1.33 (1.09–1.63)**	^**a**^**1.47 (1.04–2.07)**

Note: ^a^2 “fear of falling” variables were added (not at all concerned vs other; fairly concerned vs other)

*Abbreviations*: *CI* confidence interval, *CKD* chronic kidney disease, *GDS* geriatric depression score, *IADL* instrumental activities of daily living, *OR* odds ratio, *SPPB* short physical performance battery

## Discussion

In this paper, being old and female are predictors of falls as well as injurious falls. This results are controversial since Clemson et al. [30], found that men were at higher risk of falls while Todd and Skelton [18] found that female were at higher risk of falls. In our cohort about 38% of men and 61.8% of women experienced a fall. In line with former studies [18, 19] are our findings that women are at higher risk of falls. We also found that falls and injurious falls were related to lower physical function and self-reported disability (obtained with the SPPB Total Score, decreased IADL Score and depression). This was in agreement with Clemson and colleagues [30], who found that injurious falls in the old age, were associated with lower physical function and depression. In addition, Gunter et al. [31] suggested that physical performance tests differed between people with and without a history of falls. Similarly, to Clemson et al. [30], we found a decreased quality of life and living alone as variables that are associated with falls. While Clemson et al. [30] found that FoF did not predict injurious falls, we found that the FoF in fallers and injured fallers was significantly higher than in non-fallers and non-injurious fallers respectively.

In our regression analysis, CKD defined by eGFR did not predict falls or injurious falls. Past studies have shown controversial results. While Kistler et al. [14] and Paliwal et al. [15] demonstrated an increased odds ratio for falls in relation to CKD defined on the basis of clinical history and without reporting eGFR, a recent meta-analysis by Goto et al. [32] could not find a clear relationship between eGFR values and accidental fall risk. Nevertheless, a trend for worsening eGFR increasing accidental falls risk was observed, as well as a significant association between low eGFR and fractures resulting from accidental falls [32]. Additionally, CKD may not represent a risk factor on itself, but rather associated variables, e.g. reduced functional status, may mediate its detrimental effects [32]. The finding that patients with more severe stages of CKD are associated with coexisting conditions (e.g. IADL dependency and low SPPB score) which were found associated with falls in our analysis is in keeping with such hypothesis. Additionally, it is also important considering that present study shows cross-sectional data, and prospective analysis also including worsening CKD status as a risk factor may lead to different results.

In the SCOPE participants, about 33% of participants had experienced at least one fall and of these 35% had experienced an injurious fall, which generally resembles former studies. Falls or injurious falls are related to a decreased quality of life, and lower physical function. Therefore, it is of importance to nephrologists to keep in mind that their older CKD patients might have a higher fall risk especially their older female patients. International recommendations require all health care professionals to ask their patients 65 years and older annually about falls history or unsteadiness in balance [33]. The STEADI toolkit from the Center for Chronic Disease [34] supports and helps to identify older persons with higher risk of falling. The early identification of older persons at risk of falls is important and helps to reduce medical costs [35], and thus relieve the public health care systems. Therefore, nephrologist normally dealing with kidney function should be aware of the fall risk in their older patients and ways of treatments.

We found that prevalence of LUTS is significantly higher in fallers vs non-fallers, and such association was confirmed in all multivariable models. Current evidence about the association of LUTS and injurious falls is controversial. In a 2-years follow-up study Schluter et al. [36] showed that for men and women urinary incontinence is not a risk factor for hip fractures, but for falls. However, the association of urinary incontinence and falls was described in several studies [2, 37, 38], and urinary incontinent patients have a higher rate of falls and recurrent falls [36, 39–41]. Several studies addressing urinary incontinence included only women [38, 39, 42], whereas urinary incontinence occurs in both men and women [43]. Additionally, a systematic review by Nogouchi et al. [44] showed that urinary incontinence and LUTS was significantly associated with falls in community-dwelling older men. In our study, female gender was significantly associated with falls and injurious falls in LUTS analysis. Nevertheless, adjusting for gender did not change the strength of the observed association. We need to recognize the limited comparability of our findings to those obtained in former studies due to differences in methods for the assessment of LUTS and/or urinary incontinence. Additionally, urinary incontinence was found associated with risk of falling and fracture in community dwelling older women [39], while we included all injurious falls the patients experienced (treated and untreated injuries) not only fractures in our study. Anyway, findings from the present study suggest that increased likelihood of falls and injurious falls in relation to LUTS apply to both men and women.

### Limitations and strengths

In the present study, some limitations warrant consideration. Falls data were collected by self-reported questionnaire retrospectively, with a possible over- or underestimation of falls. This is a cross-sectional study, and prospective analysis may yield different results. Falls follow-up studies are needed to determine whether eGFR values or worsening kidney function over time may increase the risk of falls. Third, the data came from a sample that was drawn from a relatively healthy community-based population; therefore, these results cannot be generalized to frail or institutionalized older persons.

An important strength of this study is the inclusion of participants of a community-dwelling population in real-life setting. Furthermore, we considered a wide set of potential confounders. In addition, the strict differentiation between falls and injurious falls enhance current understanding and will provide further evidence over the longitudinal follow-up.

## Conclusions

LUTS may be cross-sectionally associated with increased likelihood of falls and injurious falls in community-dwelling people aged 75 or more, whereas CKD may not be associated with these outcomes. While the association between CKD and falls deserves to be prospectively investigated, treating LUTS and physical limitation might be an appropriate way to reduce risk of falling among older people, whatever is their kidney function.

## Data Availability

Data will be available for SCOPE researchers through the project website (www.scopeproject.eu).

## Abbreviations

CIRS-G Total Score: Cumulative Illness Rating Scale for Geriatrics
CI: Confidence Interval
CKD: Chronic Kidney Disease
eGFR: Estimated Glomerular filtration rate
FoF: Fear of Falling
FrPC: Fall-related psychological concern
GDS: Geriatric Depression Scale
IADL: Instrumental Activity of Daily Living
MMSE: Mini-Mental State Examination
OR: Odds ratio
SPPB: Short Physical Performance Battery
